# Up-regulated microRNAs in blastocoel fluid of human implanted embryos could control circuits of pluripotency and be related to embryo competence

**DOI:** 10.1007/s10815-025-03457-x

**Published:** 2025-03-26

**Authors:** Rosalia Battaglia, Angela Caponnetto, Carmen Ferrara, Anna Fazzio, Cristina Barbagallo, Michele Stella, Davide Barbagallo, Marco Ragusa, Maria Elena Vento, Placido Borzì, Paolo Scollo, Luca Carli, Michael Feichtinger, Evangelia Kasapi, Elias Tsakos, Simone Palini, Wojciech Sierka, Basilio Pecorino, Maria Rosaria Campitiello, Carlo Ronsini, Michele Purrello, Domenico Valerio, Salvatore Longobardi, Thomas D’Hooghe, Cinzia Di Pietro

**Affiliations:** 1 Department of Biomedical and Biotechnological Sciences, Section of Biology and Genetics “G. Sichel”, University of Catania, 95123 Catania, Italy; 2 Department of Medicine and Surgery, University of Enna “Kore”, 94100 Enna, Italy; 3 IVF Unit, Cannizzaro Hospital, Catania, Italy; 4 Wunschbaby Institut Feichtinger, Vienna, Austria; 5 EmbryoClinic IVF, Kalamaria, Thessaloniki, Greece; 6 IVF Unit, Cervesi Hospital, Cattolica, Italy; 7 Gyncentrum Sp, Żelazna 1, 40-851, Katowice, Poland; 8 Department of Obstetrics and Gynecology and Physiopathology of Human Reproduction, ASL Salerno, Salerno, Italy; 9 Department of Woman, Child and General and Specialized Surgery, University of Campania “Luigi Vanvitelli”, Naples, Italy; 10 Institute of Genetic Research (IRG), 80143 Naples, Italy; 11 Global Clinical Development, Merck Serono SpA, Rome, Italy; 12 Merck KGaA, Darmstadt, Germany; 13 Department of Development and Regeneration, Biomedical Sciences Group, KU Leuven (University of Leuven), Leuven, Belgium

**Keywords:** Blastocoel fluid, Embryo development, Embryo implantation, Human blastocyst, MicroRNAs

## Abstract

**Purpose:**

The paper aims to investigate the biological role of microRNAs secreted by preimplantation embryo into the blastocoel fluid and to detect a distinctive molecular signature for identifying embryos with the highest implantation potential.

**Methods:**

We carried on a multicenter retrospective study involving five European IVF centers. We collected 112 blastocoel fluid samples from embryos on day 5 post-fertilization, cultured individually, along with data on blastocyst grade and embryo transfer outcomes. Using a custom TLDA Array, we compared the expression levels of 89 miRNAs between 33 fluids from high-quality implanted embryos and 30 fluids from high-quality not-implanted embryos. Expression differences were assessed using SAM and t-test. Additionally, correlation and function enrichment analysis and network construction were conducted to identify the biological roles of deregulated microRNAs.

**Results:**

We identified six up-regulated microRNAs in the blastocoel fluid from implanted embryos, significantly and positively correlated across all samples (*r* ≥ 0.7; *P* ≤ 0.05). They could take part in pluripotency circuits, regulating and being regulated by transcription factors associated with stemness, cell growth, and embryo development. The ROC curve analysis confirmed the potential of these miRNAs as implantation classifiers.

**Conclusion:**

The six miRNAs up-regulated in blastocoel fluid from implanted embryos may represent a functional molecular signature for evaluating blastocyst quality and identifying the most competent embryos. Their evaluation associated with non-invasive preimplantation genetic testing, integrating epigenetic and genomic analyses, could enhance implantation grade and allow for identification of the euploid embryo not able to implant.

**Supplementary Information:**

The online version contains supplementary material available at 10.1007/s10815-025-03457-x.

## Introduction

On the 5th–6th day after fertilization, the human embryo reaches the blastocyst stage and is ready for implantation. Multiple factors, including embryo competence, endometrial receptivity, and the proper dialog between the embryo and maternal tissues, influence this process [1]. Moreover, the quality of gametes and the proper progression of cellular and molecular events from the zygote to the blastocyst stage are critical for determining embryo quality and, eventually, pregnancy success [2]. Understanding molecular mechanisms occurring during this period is crucial for advancing basic research and improving the success of in vitro fertilization (IVF) cycles.

Despite the advancements, the success rate of IVF cycles remains relatively low, at approximately 20%. However, relying exclusively on morphological evaluation of the embryo is inadequate to ensure a successful pregnancy and the birth of healthy babies [3, 4].

Genetic defects in embryos contribute significantly to implantation failures [5], prompting the development of strategies like preimplantation genetic testing (PGT) to enhance pregnancy rates and live births in IVF cycles [6]. However, concerns regarding the invasive nature of current biopsy methods and mosaicism-associated risks persist [7].

Alternatively, researchers are turning their attention to the potential of utilizing cell-free DNA found in both spent culture medium (SCM) and blastocoel fluid (BF) for genetic testing [7]. Utilizing SCM or BF as a source for assessing specifically embryonic DNA offers advantages over trophectoderm (TE) biopsy as it avoids the need to collect and potentially compromise embryonic cells [8]. Blastocentesis, in comparison to the collection of SCM, is a more invasive procedure that requires a higher level of skill and time from the embryologist. Moreover, it exposes the embryo to suboptimal environmental conditions outside the incubator [9]. On the other hand, analysis of BF could better reflect embryo status, avoiding the risk of contamination with somatic female cells or sperms [8].

Cell-free embryo DNA analysis provides a comprehensive view of the embryo’s genomic context. Additionally, the possibility of using other biomarkers to evaluate the competence of the embryo before transfer could provide an epigenetic perspective that may not be detected by genomic analysis [10].

In the last few years, extracellular vesicles (EVs) have been isolated from BF and SCM of embryos obtained from IVF cycles [11, 12]. Among the different molecules of EV cargo, microRNAs (miRNAs) are key components [13–15]. They can control multiple cellular pathways by regulating gene expression at the post-transcriptional level [16–18]. Recent studies have highlighted the developmental dynamics of small non-coding RNA expression from day 3 (E3) to day 7 (E7) of human embryo development, profiling and quantifying all major small non-coding RNA (sncRNA) biotypes. Notably, miRNAs appear to be developmentally regulated during the E3 to E7 window of compaction and blastulation, underscoring their potential roles in potency, lineage specification, and maintenance [19]. Furthermore, analyses of miRNA clustering and targeting highlighted their complex involvement in the transition from totipotency to pluripotency, particularly within the ICM and TE lineages [19]. In recent years, scientific interest has been focused on their potential use as non-invasive biomarkers of embryo quality. Several studies have compared miRNA expression profiles in SCM between implanted or not-implanted and euploid or aneuploid embryos. In 2016, Capalbo et al. found two miRNAs up-regulated in SCM from euploid implanted embryos compared with not-implanted ones [20]. In 2021, Wang et al., using RNA sequencing, identified six differentially expressed miRNAs in SCM, between implanted and not-implanted embryos [21]. In 2022, Esmaeilivand and colleagues, employing real-time PCR, demonstrated the up-regulation of miRNAs in BF from aneuploid embryos compared to euploid ones [22].

However, to the best of our knowledge, no paper has compared miRNA profiles in BF in relation to embryo outcome and investigated their biological role in humans.

In 2019, our research group isolated exosomes from BF and analyzed their miRNA cargo, finding their involvement in pluripotency, cell reprogramming, epigenetic modification, intercellular communication, cell adhesion, and cell fate [11]. BF miRNAs may act as paracrine or autocrine messengers, sending different signals among blastocyst cells, maintaining the stemness of the inner cell mass (ICM), and driving TE differentiation [11, 19]. If these molecules play an important role in the early stages of embryonic development, it is rational to believe that their altered expression can influence embryo competence, making them potential molecular markers of embryo quality.

In this study, we collected 112 BF samples from five IVF centers and evaluated the expression of 89 previously identified miRNAs [11]. Upon receiving the data on transferred embryos’ outcomes, we compared miRNA profiles between implanted and not-implanted embryos. We identified specific miRNAs whose deregulated expression in embryos with low implantation potential could affect important biological pathways involved in the early stages of embryonic development. We propose that the identified miRNAs may serve as a molecular signature of embryo quality. Assessing their expression levels before embryo transfer into the uterus could predict implantation potential, thereby improving the success rates of IVF cycles.

The knowledge of biological aspects of human pre-implantation development might provide not only insights into human developmental biology and common birth defects but also potential benefits for reproductive health and improvements in regenerative medicine.

## Materials and methods

### Ethics statement

The study was approved by the Ethics Committee Catania 1 (n. 178–2018-CA). Informed consent was obtained from the couples, and the experiments were performed following the principles set out in the World Medical Association Declaration of Helsinki.

### Sample collection

One hundred and twelve BF samples were collected by 5 IVF centers: Cannizzaro Hospital IVF Unit, (Catania, Italy), Embryoclinic IVF Unit (Kalamaria, Greece), Gyncentrum-Klinica Leczenia Niepłodnościi Diagnostyki Prenatalnej (Katowice, Poland), Wunschbaby Institut Feichtinger (Vienna, Austria), and Ospedale Cervesi Cattolica (Cattolica, Italy), according to a previously published protocol [11]. Blastocoel fluid samples were retrieved from embryos on day 5 post-fertilization, cultured individually in single drops, along with data on blastocyst grade and embryo transfer outcomes.

All blastocysts underwent blastocentesis, artificial shrinkage, and subsequent vitrification. The blastocoel can impede cryoprotectant permeation, increasing the risk of ice crystal formation and cellular damage. To mitigate this, artificial shrinkage was performed by aspirating the blastocoel fluid immediately before vitrification, thereby collapsing the cavity [23] This technique has been reported to enhance post-warming survival rates and improve pregnancy outcomes [24]. The vitrification protocol employed a brief exposure to small volumes of highly concentrated solutions containing a combination of two permeating cryoprotectants, dimethyl sulfoxide (DMSO) and ethylene glycol (EG), along with 0.5 M sucrose as an external cryoprotectant. Additionally, a rapid cooling and warming rate exceeding 10,000 °C/min was utilized to minimize ice crystal formation. A closed vitrification system was selected to prevent direct contact with liquid nitrogen, ensuring both sterility and optimal cryopreservation efficiency [25, 26]. After warming, the blastocysts were then cultured for 3–4 h in the incubator to allow re-expansion. No delay was recorded compared to the expected time. The quality was always the same as before vitrification.

Morphological classification of human blastocysts was conducted according to Gardner’s classification system [3]. In total, we collected and performed the miRNA profiling of 112 BF from blastocysts of different grades. Among them, we had to focus our attention on 63 high-quality embryos being the only ones transferred in utero and for which we had available data on implantation. The characteristics of the samples are described in Table 1.

**Table 1:** Information on BF samples analyzed

Characteristics	Implanted group	Not-implanted group	Total	*P*-value
**Overall**	*N* = 33	*N* = 30	*N* = 63	
Blastocyst grade	*** N*** 3AA 6 3AB 1 4 AA 25 4AB 1	*** N*** 3AA 7 3AB 1 3BA 1 3BB 1 4AA 14 4AB 1 4BA 2 5AA 3		
Individual embryo culture	100%	100%		
Patient age—mean (SD)	32.1 ± 5.2	31.9 ± 6.7		*0.88262514*

After blastocentesis, BF samples were diluted in 5 µL of RNase-free water into a 0.2-mL sterile PCR tube, stored at − 80 °C, and sent to the Biomedical and Biotechnological Department laboratories in Catania, Italy, for molecular analysis.

### Analysis of miRNAs in blastocoel fluid

#### Design and analysis of the custom TLDAs

In our previously published paper, we identified 89 miRNAs in BF, by using TaqMan Low-Density Array (TLDAs) technology (panel A) (Applied Biosystem) able to analyze 384 miRNAs [11]. The 89 expressed miRNAs were used to design custom TLDAs (Applied Biosystem) able to analyze the BF from four different samples in a single experiment. In Table S1, we reported the custom cards scheme.

As previously reported, RNA from BF samples was extracted by thermolysis, incubating it for 1 min at 100 °C to release nucleic acids [11]. Subsequently, 5 µL of each BF sample was directly reverse-transcribed and pre-amplified in a final volume of 15 µL and 50 µL, respectively, according to the manufacturer’s guidelines. Quantitative RT-PCR reactions were performed on a QuantStudio 7 Flex Real-Time PCR System (Applied Biosystems) as follows: 95 °C for 10 min, followed by 40 amplification cycles of 95 °C for 15 s and 60 °C for 1 min. QuantStudio Real-Time PCR software v1.3 (Applied Biosystems by Thermo Fisher Scientific) was used for data analysis [11]. Only miRNAs having Ct values higher than 15 and lower than 35, showing optimal amplification plots, and detected in at least 50% of samples were considered as expressed and subjected to statistical analysis. Undetermined Ct values were assigned a value of 40. miRNAs that did not meet these criteria were excluded from further analysis, as they might represent unreliable or inconsistent data. Data normalization was performed by using the U6 gene as endogenous control. To ensure robustness and minimize technical artifacts, U6 probes were spotted twice per sample during the card design, yielding a total of eight replicates per plate. To quantitatively assess the stability of U6, we employed NormFinder, a widely recognized algorithm that evaluates expression variation across samples and experimental groups to identify the most stable reference genes [27]. NormFinder assigned U6 a stability value of 0.005. Consequently, the mean expression level of U6 was calculated and utilized for miRNA normalization. In Table S2, the ΔCt values are reported.

#### Expression data analysis

Differentially expressed (DE) miRNAs were identified by significance analysis of microarrays (SAM) tests (Tusher, minimum S value, the 5th, 50th, and 90th percentiles), using MeV (Multi experiment Viewer v4.8.1) software. The SAM approach is a robust statistical technique introduced by Tusher, Tibshirani, and Chu (2001) [28] to identify significantly differentially expressed genes in high-throughput biological data. SAM assigns each gene a score statistic that quantifies the change in expression between conditions, normalized by an estimate of its variability. This score helps detect genes whose changes in expression are unlikely to have occurred by chance. To assess the statistical significance of these scores, SAM uses permutation testing. In this approach, sample labels are shuffled (e.g., implanted vs. not-implanted) 100 times to simulate the null hypothesis and build a null distribution for the scores.

A two-class unpaired SAM test was applied to ΔCt values of implanted and not-implanted BF samples, using a *P*-value based on 100 permutations; imputation engine: K-nearest neighbors (10 neighbors). A false discovery rate (FDR) < 0.05 was used as a correction for multiple comparisons. DE miRNA expression changes were calculated by applying the 2^−ΔΔCT^ method according to a previously published paper [29]. Additionally, an unpaired t-test was also applied. Statistical significance was assessed by setting the *p*-value cut-off at ≤ 0.05.

### Genomics of BF miRNAs

The mature miRNA sequences were retrieved from MirBase (http://mirbase.org/). The chromosome and nucleotide positions were determined using the UCSC Genome Browser (https://genome.ucsc.edu/) and the database Gene of NCBI (http://www.ncbi.nlm.nih.gov/gene). Multiple sequence alignment was carried out using the Clustal Omega program (https://www.ebi.ac.uk/Tools/msa/clustalo/) to identify the consensus among miRNA sequences. Finally, CLC Sequence Viewer v 6.0 was used for analyzing conserved motifs among the different consensus strings of clustered miRNAs.

### miRNA-miRNA expression correlation

Pearson’s correlation analyses were computed among the normalized data of differentially expressed (DE) miRNAs for the implanted-not-implanted comparison, by GraphPad Prism v8.01. Significant correlations were selected at an *r* cut-off value ≥ 0.7. Statistical significance was established at a *p*-value ≤ 0.05.

### miRNA function enrichment analysis

Gene Ontology (GO) and Kyoto Encyclopedia of Genes and Genomes (KEGG) analyses were performed to explore the potential biological roles of the six DE miRNAs. We queried *Diana-miRPath v3.0* (https://dianalab.e-ce.uth.gr/html/mirpathv3/index.php?r=mirpath) selecting for validated mRNA targets retrieved from Tarbase 7.0. DE miRNAs were analyzed for GO enrichment in terms of the biological process (BP) categories applying a *p*-value cut-off of 0.05. For KEGG analysis, the false discovery rate (FDR) method was applied to identify the signaling pathways with a threshold of significance defined by *p* ≤ 0.05 and a microT threshold of 0.8.

### Networks of transcription factors-miRNA-mRNA interactions

To investigate the potential molecular role of DE miRNAs, their mRNA targets were retrieved from the Homo Sapiens catalog by *miRTarbase 8.0* database (https://mirtarbase.cuhk.edu.cn/~miRTarBase/miRTarBase_2022/php/index.php) selecting only validated targets by functional miRNA-target interaction. Interaction network diagrams of DE miRNA-targets were then constructed by *Cytoscape software 3.9.0*. The resulting networks were analyzed using the *Cytoscape app CytoHubba* (Version 0.1). The nodes were ranked according to the degree (Deg) scoring method. Additionally, the *TransmiR v2.0* database (http://www.cuilab.cn/transmir) was used to identify the relationship between DE miRNAs and the transcription factors (TFs) that are involved in pluripotency regulation. Only targets validated and common to at least two miRNAs were considered*.* The interaction network diagram of TF-miRNA-target genes was constructed by *Cytoscape software* 3.9.0.

### Receiver operator characteristic curve analysis

The ΔCt values of the six correlated DE miRNAs from the comparison implanted-not implanted served as input data to perform a classical univariate receiver operator characteristic (ROC) curve analysis using MedCalc Statistical software version 22.001. Moreover, logistic regression analysis was employed to calculate the ROC curves of the six correlated DE miRNAs considered together, and k-fold cross-validation was performed. To test the suitability of selected miRNAs as biomarkers, the optimal cut-off point for each ROC curve was determined by calculating the Youden index (J), a common summary measure used to evaluate a biomarker’s ability to classify disease status [30, 31].

## Results

### miRNA expression profile in blastocoel fluid according to blastocyst implantation

**Fig. 1:**
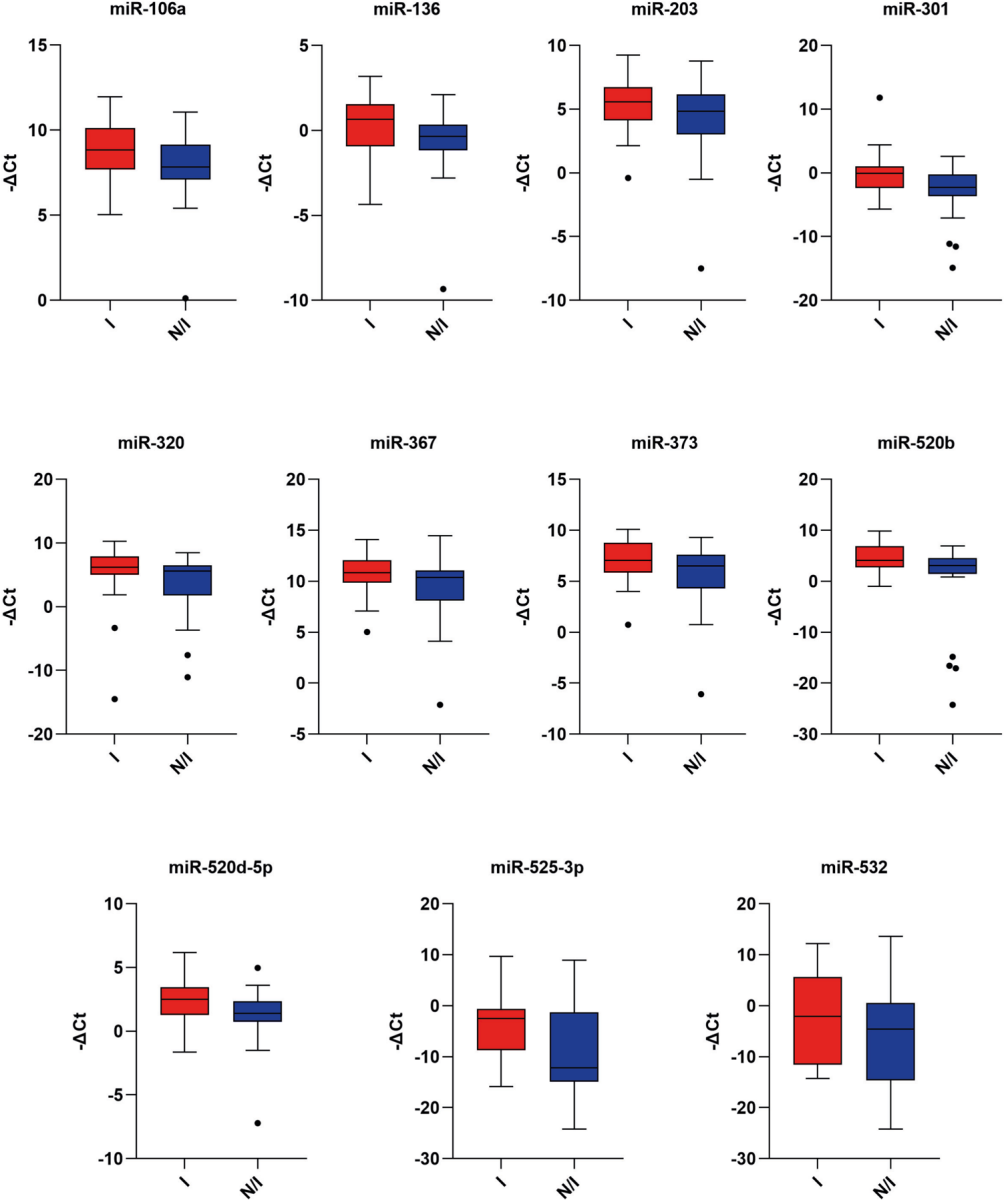
Box and whisker plots showing the relative expression of differentially expressed (DE) miRNAs. miR-106a (*p*-value = 0.02), miR-136 (*p*-value = 0.03), miR-203 (*p*-value = 0.03), miR-301 (*p*-value = 0.01), miR-320 (*p*-value = 0.09), miR-367 (*p*-value = 0.03), miR-373 (*p*-value = 0.02), miR-520b (*p*-value = 0.01), miR-520d-5p (*p*-value = 0.01), miR-525-3p (*p*-value = 0.03), and miR-532 (*p*-value = 0.02) are significantly up-regulated in implanted blastocysts. The analysis was conducted on 33 BF from implanted embryos and 30 BF from not implanted embryos. Expression data are represented as –ΔCt values normalized against U6. Black dots represent outlier values. I, implanted blastocysts; N/I, not implanted blastocysts

The basic information on BF samples analyzed in the study and the statistical differences between the two groups (implanted and not-implanted embryos) are presented in Table 1. Despite all embryos receiving high grading scores based on morphological assessment, differences in miRNA expression were significantly associated with successful implantation and were not related to blastocyst grade. miRNA expression profile analysis comparing implanted and not-implanted embryos revealed eleven statistically significant up-regulated miRNAs (miR-106a, miR-136, miR-203, miR-301, miR-320, miR-367, miR-373, miR-520b, miR-520d-5p, miR-525-3p, and miR-532) (Fig. 1).

### Genomics of the eleven DE BF miRNAs

**Fig. 2:**
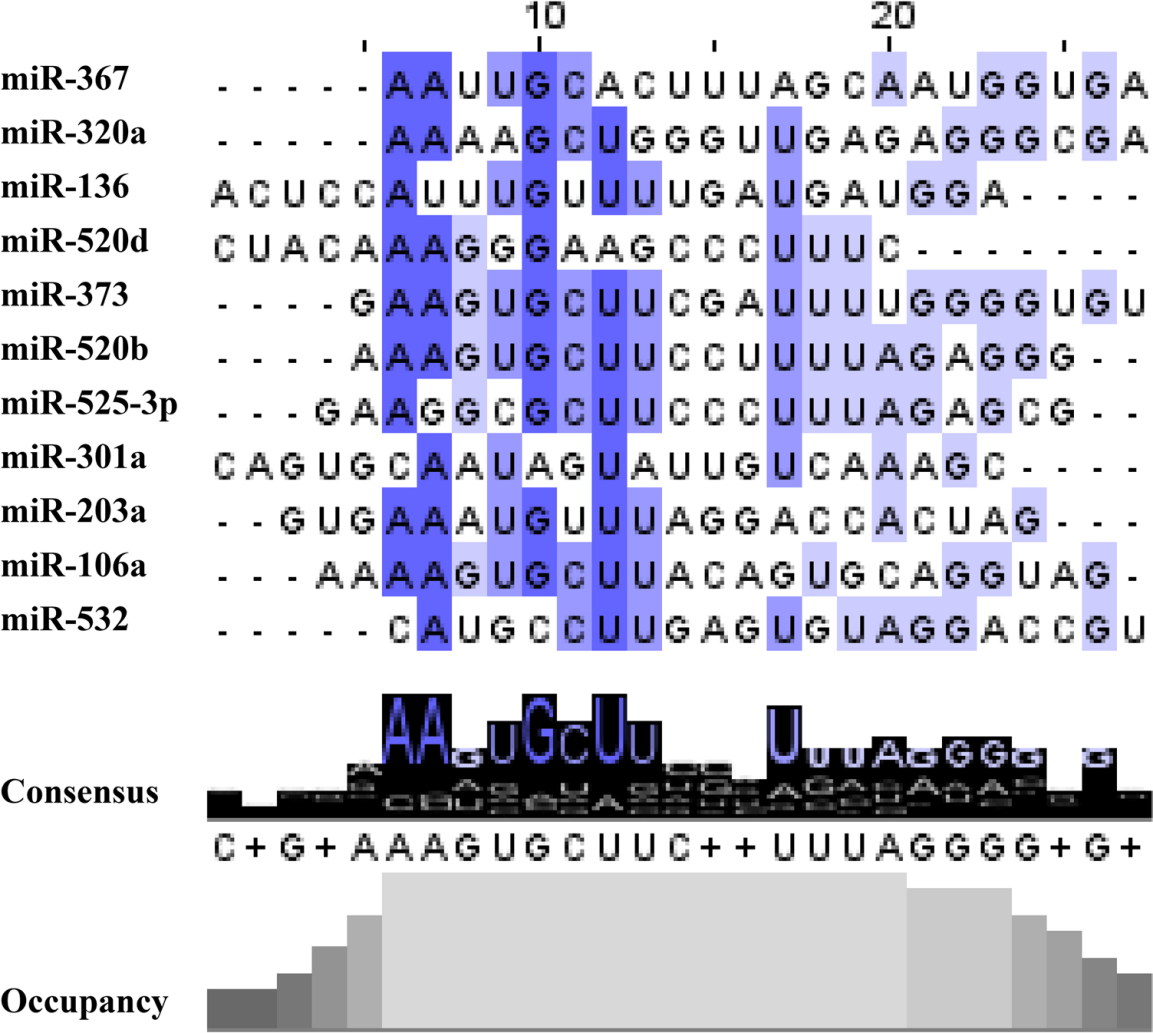
Consensus clustering and frequency of nucleotide conservation for up-regulated blastocoel fluid (BF) miRNAs

Most of the DE miRNAs are part of different families lying in independent genes. However, miR-520b-3p, miR-520d-5p, and miR-525-3p belong to the same chromosome cluster (Table 2). Alignment of the mature sequences of the eleven up-regulated miRNAs revealed full or partial conservation of the embryo miRNA motif AAGUGC at the 5’-end of the functional mature miRNA (Fig. 2).

**Table 2:** Genomics of DE miRNAs

MicroRNA	Chromosome	Position (GRCh38.p14)	Cluster
miR-106a-5p	Xq26.2	134,170,198–134170278	miR-106a/miR-18b/miR-20b/miR-19b-2/miR-92a-2/miR-363
miR-136-5p	14q32.2	100,884,702–100884783	miR-493/miR-337/miR-665/miR-431/miR-433/miR-127/miR-432/miR-136
miR-203a-3p	14q32.33	104,117,405–104117514	miR-203a/miR-203b
miR-301a-3p	17q22	59,151,136–59,151,221	
miR-320a-3p	8p21.3	22,244,966–22,245,037	
miR-367-3p	4q25	112,647,874–112,647,941	miR-302b/miR-302c/miR-302a/miR-302d/miR-367
miR-373-3p	19q13.42	53,788,705–53788773	miR-371a/miR-371b/miR-372/miR-373
miR-520b-3p	19q13.42	53,701,227–53701287	miR-512–1/miR-512–2/miR-1323/miR-498/miR-520e/miR-515–1/miR-519e/miR-520f/miR-515–2/miR-519c/miR-1283–1/miR-520a/miR-526b/miR-519b/miR-525/miR-523/miR-518f/miR-520b/miR-518b/miR-526a-1
miR-520d-5p		53,720,096–53720182	
miR-525-3p		53,697,533–53,697,617	
miR-532-5p	Xp11.23	50,003,148–50003238	miR-532/miR-188/miR-500a/miR-362/miR-501/miR-500b/miR-660/miR-502

### miRNA-miRNA expression correlations

**Fig. 3:**
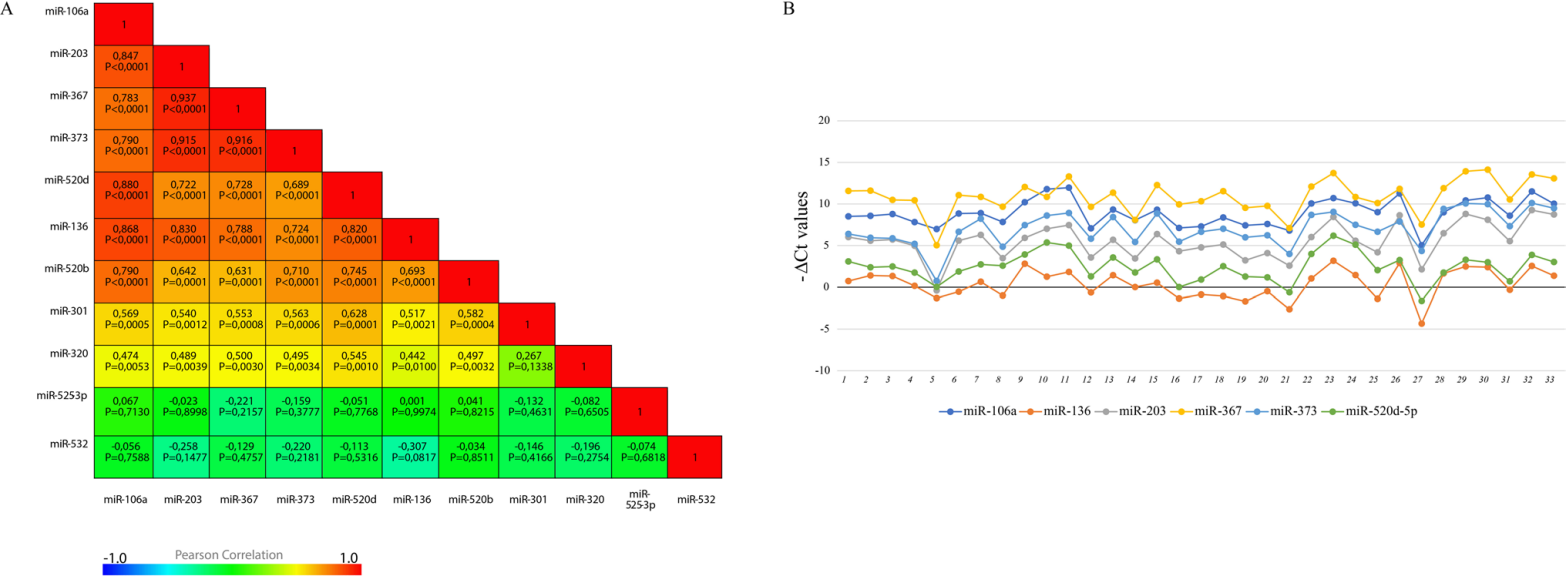
miRNA-miRNA correlation in implanted embryos. **A **Correlation matrix with Pearson correlation values and *p*-values for implanted embryos. **B **Expression trend of the six correlated miRNAs, in implanted embryos

**Fig. 4:**
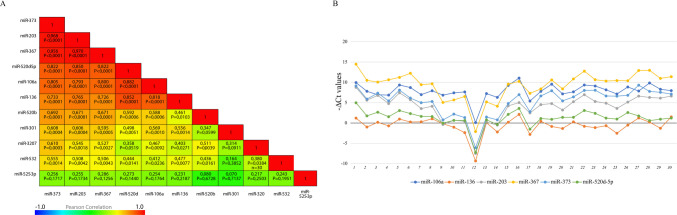
miRNA-miRNA correlation in not-implanted embryos. **A **Correlation matrix with Pearson correlation values and *p*-values for not-implanted embryos. **B **Expression trend of the six correlated miRNAs in not-implanted embryos

The correlation matrix showed significantly positive correlations with *r* ≥ 0.7 among miR-203, miR-367, miR-373, miR-136, miR-106a, and miR-520d-5p, both for implanted and not-implanted embryos (Figs. 3A and 4A). MiR-520b-5p showed a significantly positive correlation with *r* ≥ 0.7 with miR-106, miR-373, and miR-520d only in implanted embryos (Fig. 3a). MiR-301 and miR-320 showed a lower correlation with *r* < 0.6, not always statistically significant, with the other miRNAs, while miR-525 and miR-532 did not show any significant correlation. The six correlated miRNAs showed similar expression trends in implanted and not-implanted embryos (Figs. 3B and 4B).

### Function enrichment analysis of six co-expressed miRNAs

**Fig. 5:**
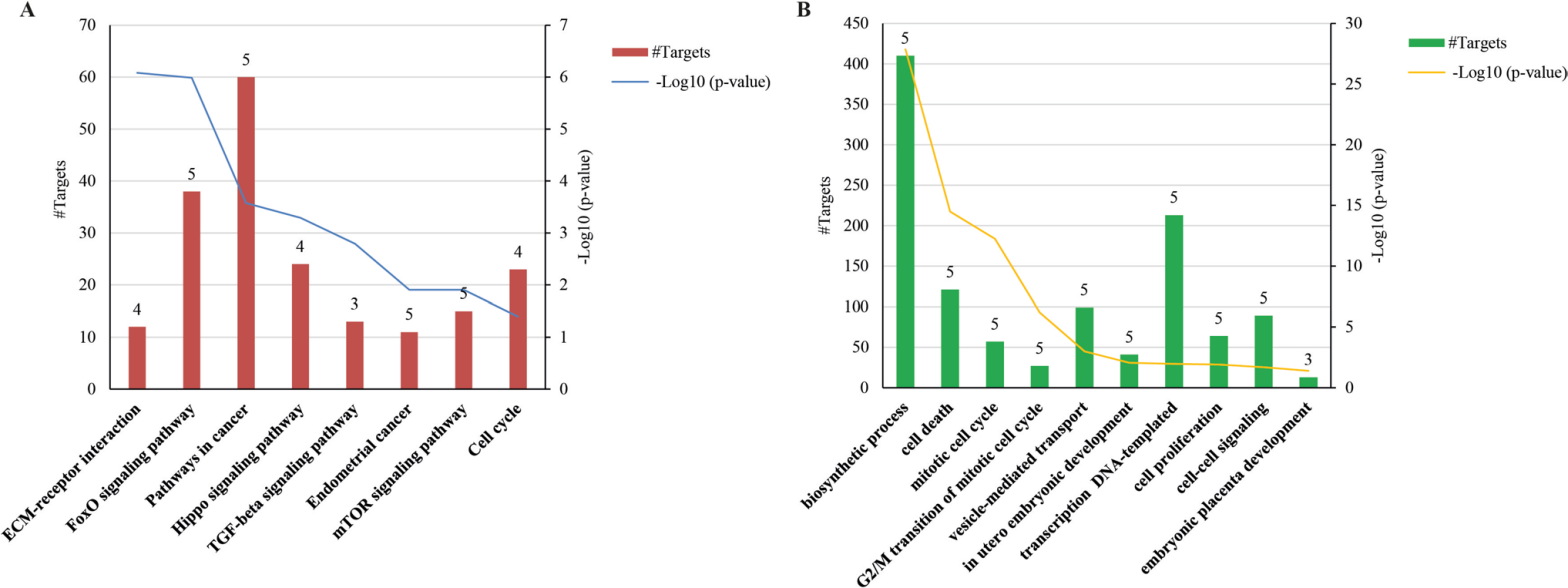
Kyoto Encyclopedia of Genes and Genomes (KEGG) and Gene Ontology (GO) analyses for the six correlated miRNAs. **A** Illustrates the significant KEGG pathways, cell cycle regulation, cell proliferation, and pluripotency regulated by the six correlated miRNAs. The results are sorted by the number of miRNA-target genes in each pathway (y-axis), the number of miRNAs involved in the regulation of each pathway (above red bars), and the significance as − log10 *P* value (secondary y-axis). **B** Displays selected Gene Ontology (GO) terms for biological processes (BPs). The results are sorted by the number of miRNA genes in each BP (y-axis), the number of differentially expressed (DE) miRNAs involved in the regulation of each BP (above green bars), and the significance as − log10 *P* value (secondary y-axis)

The six miRNAs up-regulated in BF of implanted blastocysts were predicted to exert significant control over pathways associated with preimplantation embryo development and stemness. These pathways include the ECM-receptor interaction, FoxO signaling pathway, pathways in cancer, Hippo signaling pathway, TGF-beta signaling pathway, endometrial in cancer, mTOR signaling pathway, and cell cycle. Specifically, five out of six identified miRNAs regulate the FoxO signaling pathway, endometrial cancer, and mTOR signaling pathway. Additionally, three identified miRNAs influence the TGF-beta signaling pathway (Fig. 5A). No annotated targets for miR-203a-3p were identified in Diana-miRPath, the database used to retrieve BPs and KEGG pathways. A detailed list of all the target genes involved in each pathway is reported in Table S3.

GO analysis revealed that five of the identified miRNAs, except miR-203a-3p (as previously reported), are involved in the regulation of several BPs (Fig. 5B). The most significant include biosynthetic process (involving 5 miRNAs and 410 targets), cell death (5 miRNAs and 121 targets), mitotic cell cycle (5 miRNAs and 57 targets), the transition of the mitotic cell cycle (5 miRNAs and 27 targets), vesicle-mediated transport (5 miRNAs and 99 targets), in utero embryonic development (5 miRNAs and 41 targets), transcription DNA templated (5 miRNAs and 213 targets), cell proliferation (5 miRNAs and 64 targets), cell–cell signaling (5 miRNAs and 89 targets), and embryonic placental development (3 miRNAs and 13 targets) (Fig. 5B).

### Networks of TF-miRNA-mRNA

**Fig. 6:**
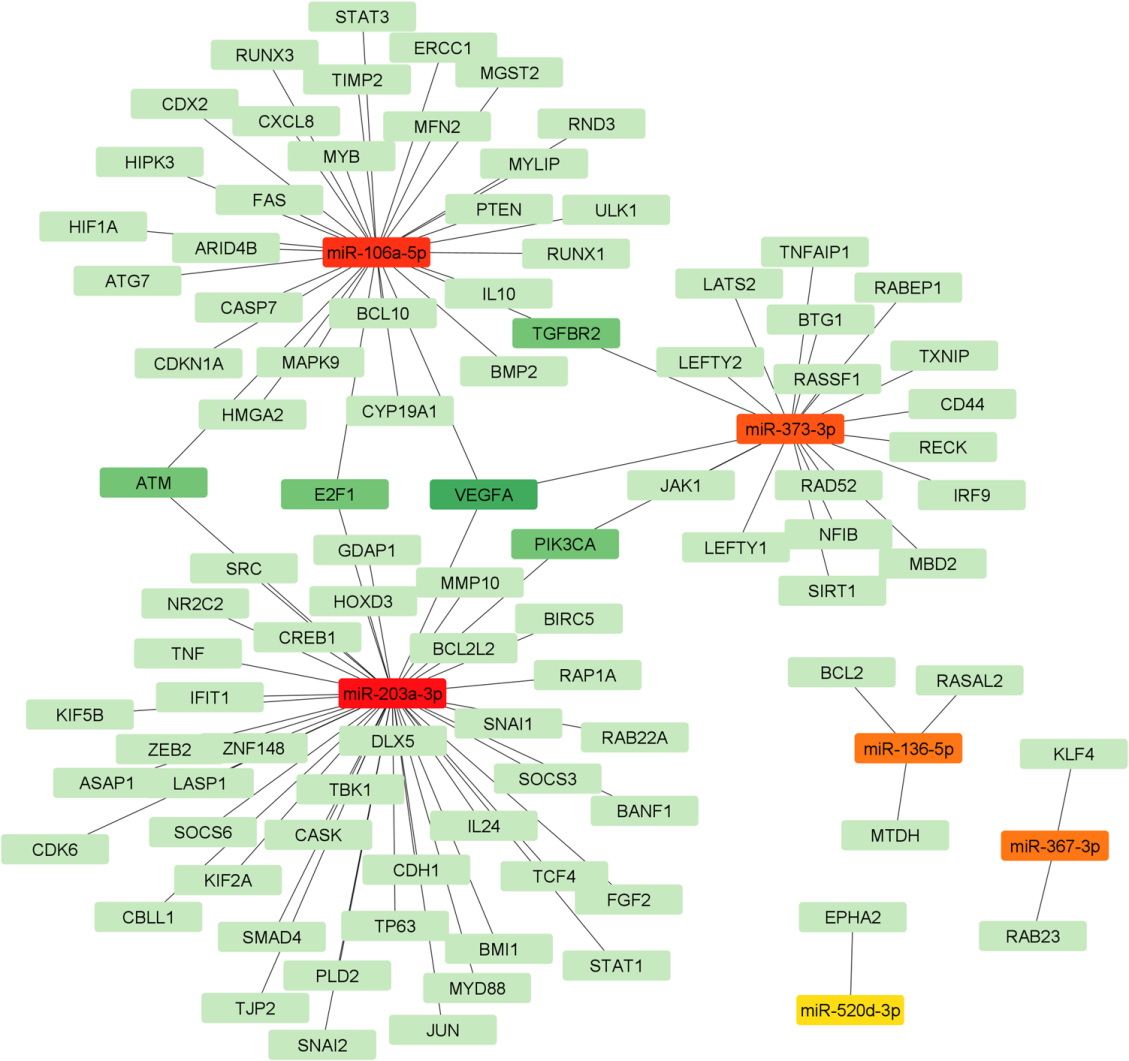
Regulatory network of miRNA-mRNA interactions in implanted blastocysts. The subgraphs show the 100 top-ranked nodes, determined using the degree scoring method. MiRNAs are depicted with a color scheme indicating their centrality, ranging from red (high centrality) to yellow (low centrality). The nodes representing mRNA targets are also displayed based on their centrality, ranging from dark green (high centrality) to light green (low centrality)

The analysis of miRNA-mRNA interactions in implanted blastocysts generated regulatory networks, with miR-203a-3p, miR-106a-5p, and miR-373-3p as the central hubs (Fig. 6). In detail, miR-203a-3p, miR-106a-5p, and miR-373-3p ranked first, second, and third with a score of 44, 31, and 19, respectively (Fig. 6). Moreover, the three miRNAs, sharing several mRNA targets, are part of the main network. Conversely, miRNA-136, miR-367, and miR-520d are not part of the main network and regulate a smaller number of not-shared mRNAs (Fig. 6).

**Fig. 7:**
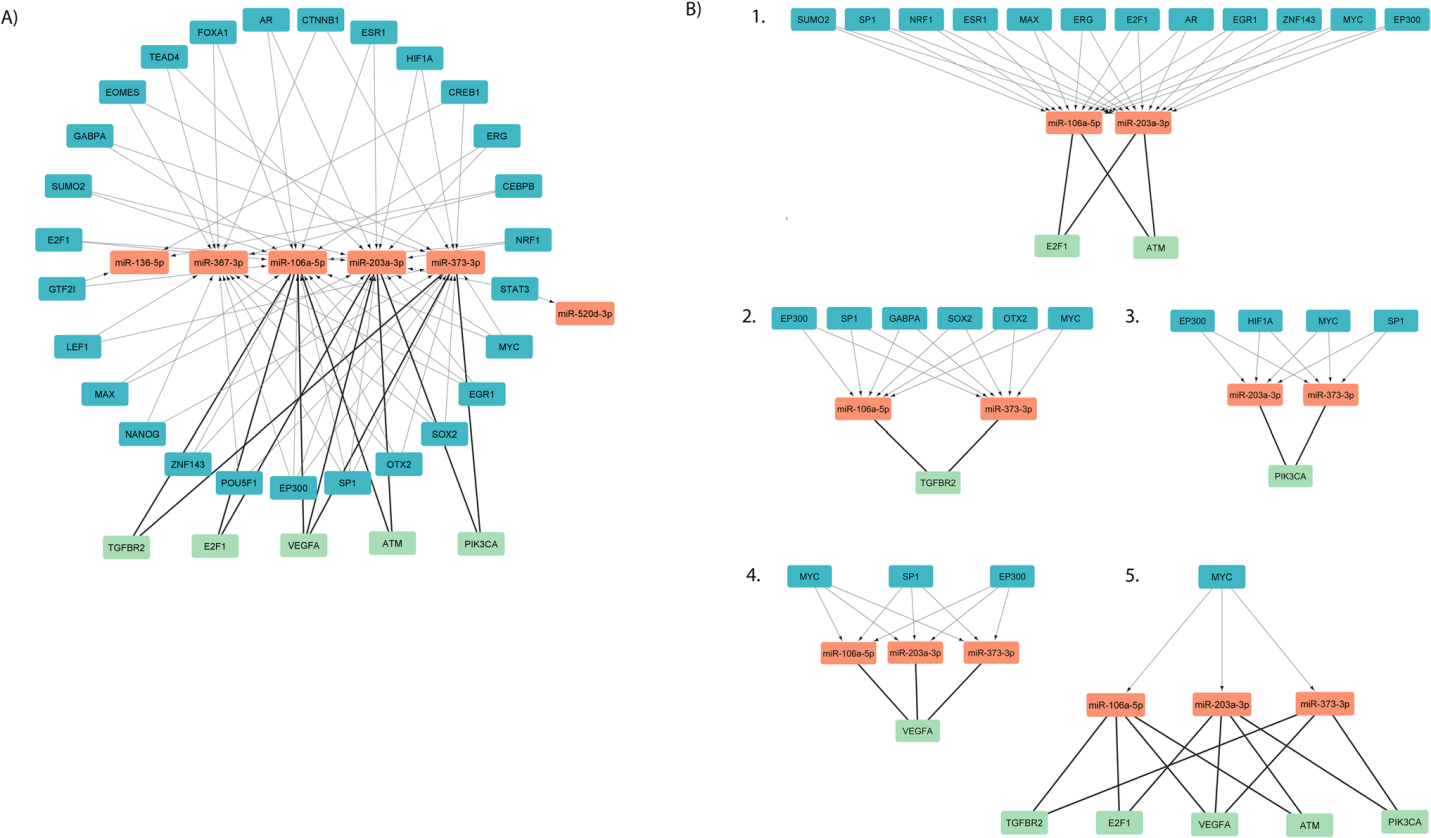
Interaction-network diagram of TF-miRNA-target genes. **A** Orange rectangles represent the miRNAs, blue rectangles the transcription factors (TFs) regulating miRNA expression, and green rectangles indicate the miRNA-target genes. **B** In the panel, we highlight five subnetworks

The analysis of six correlated miRNAs and their relationship with key transcription factors involved in pluripotency revealed that different miRNAs are regulated by the same transcription factor (Table S4). Moreover, these miRNAs are also found to share common target mRNAs. Detailed TF-miRNA-mRNA interactions are reported in Fig. 7a and supplementary table (Table S5). Figure 7b highlights regulatory loops that underscore the role of miRNAs in pluripotency circuits.

### ROC curve analysis

**Fig. 8:**
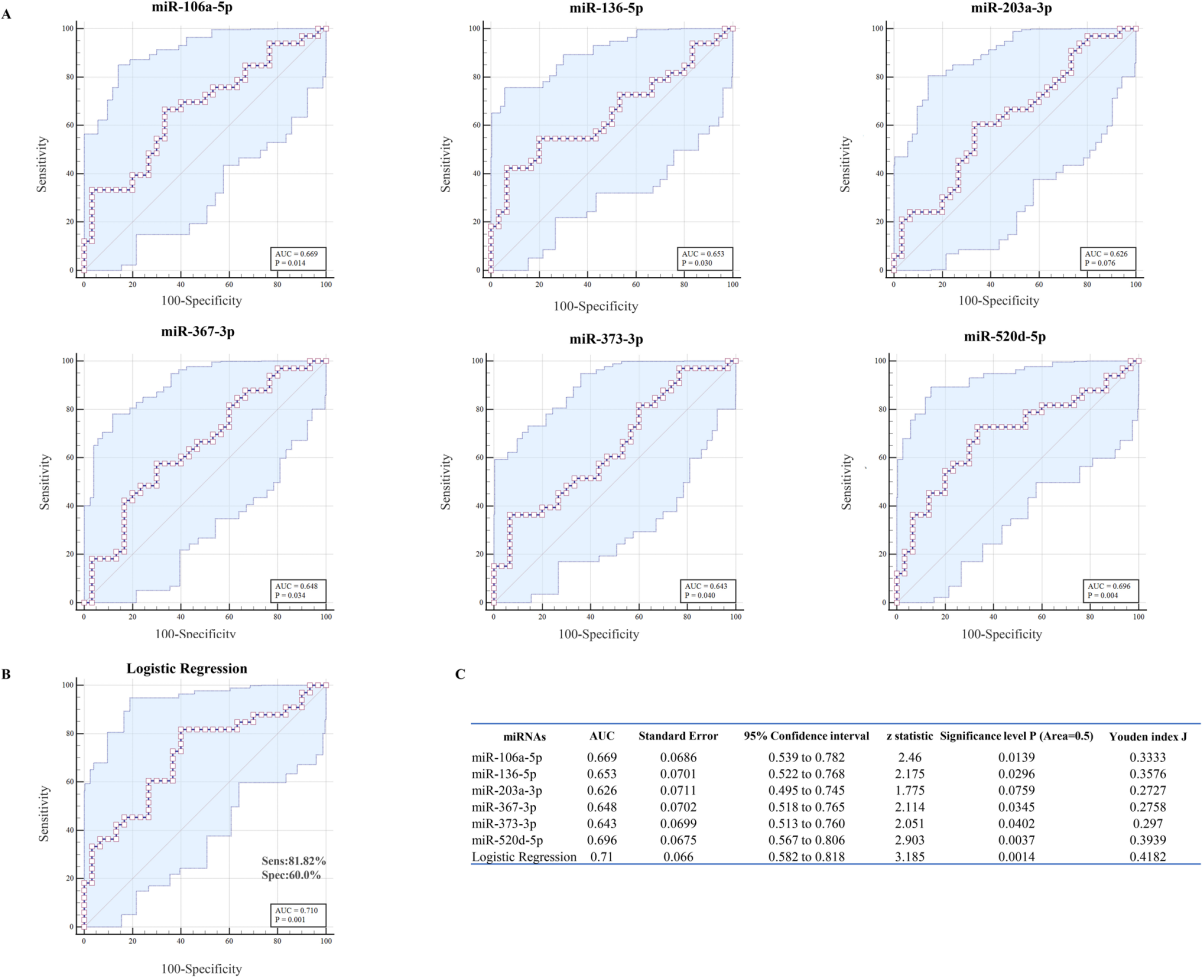
BF miRNAs as potential biomarkers of embryo implantation. **A** Classical univariate ROC curve analyses for the comparisons of implanted vs not-implanted embryos, focusing on dysregulated miR-106a, miR-136, miR-203, miR-367, miR-373, and miR-520d. Each point on the ROC curves represents a sensitivity/specificity pair. AUC, area under the ROC curve, and *P*-values are shown. **B** Logistic regression model construction. The ROC curves of six correlated miRNAs considered together were calculated using the logistic regression analysis. The sensitivity, the specificity, and AUC parameters for distinguishing between implanted and not-implanted embryos are reported. **C** ROC curve statistical analysis is shown

Univariate ROC curve analysis confirmed the reliability of the six correlated miRNAs as biomarkers of implantation. miRNA up-regulation in BF can discriminate between the implanted and not-implanted blastocysts. The univariate ROC plots revealed an area under the curve (AUC) of about 0.7 with a significant *p*-value for five of the six correlated miRNAs (Fig. 8A, C). The ROC curve analysis showed that only miR-203, with an AUC of 0.626, did not reach statistical significance (Fig. 8A).

**Fig. 9:**
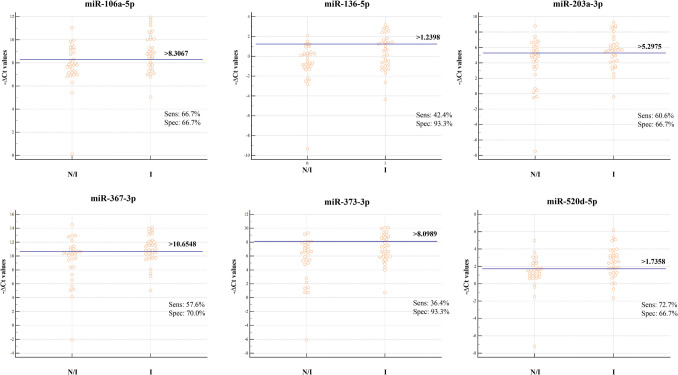
Criteria for embryo implantation prediction. The graphs show the distribution of ΔCt values of all 63 analyzed samples. ΔCt criterion divides I (implanted) from NI (not implanted) embryos, enabling accurate discrimination

Furthermore, logistic regression of ROC curves for the six correlated miRNAs showed that the diagnostic accuracy of the individual miRNAs improved when considering all six simultaneously (Fig. 8B). Logistic regression analysis yielded an AUC of 0.710 (*p* = 0.001) for distinguishing between implanted and not-implanted embryos, with 81.8% sensitivity and 60% specificity (Fig. 8B). The 5 k-fold cross-validation confirmed the results of the logistic regression analysis, yielding a mean AUC of 0.667 with a statistical significance level of 0.0157. Additionally, ΔCt value cut-offs corresponding to the sensitivity/specificity pair with the Youden index J are also provided (Fig. 9).

## Discussion

Since 2004, the crucial role of miRNAs in regulating the self-renewal and pluripotency of human embryonic stem cells (hESCs) has been well recognized [32]. During early embryonic development, miRNA expression is induced by the pluripotency factors such as SRY-box transcription factor 2 (Sox2), POU class 5 homeobox 1 (POU5F1), and Nanog, which, in turn, are regulated by these miRNAs. miRNAs can control the fate of ESCs by directly targeting the 3′ UTR or binding to the coding sequence of pluripotency factors, modulating their expression [32].

If miRNAs play an essential role inside the embryo cells, it is reasonable to expect that changes in their expression influence embryo quality, accordingly, their evaluation in BF could provide useful information on the implantation potential of the blastocyst. Moreover, BF may more accurately reflect the embryo secretome compared to the culture medium, which can be influenced by various external factors such as cumulus cells, different components of the medium, and paternal RNA (in FIVET cycles) [33–36]. In BF of implanted embryos, we found 11 up-regulated miRNAs that can be considered “embryo miRNAs” (Fig. 1). They share homology with the common hexamer seed ′AAGUGC (Embryo motif) ranging from 100 to 43% (Fig. 2), and miR-106a, miR-367 miR-373, miR-520b, miR-520d, and miR-525 have already been described belong the embryo’s miRNA family [37–39]. Interestingly, miR-106a, miR-367, and miR-373 were found to be up-regulated in TE cells of euploid embryos compared to aneuploid ones [40]. The clusters miR-371/373 and miR-302/367 have been shown to play a pivotal role in regulating pluripotency by modulating key transcriptional networks and cellular processes, including cell cycle regulation and differentiation. Interestingly, miR-373 and miR-367 are particularly enriched in trophectoderm cells and are associated with the regulation of genes critical for blastocyst development, reinforcing their relevance to embryonic competence and implantation potential [19].

A 2018 study identified 50 protein-encoding genes, termed the “essentialome,” that are critical for the growth and survival of hPSCs [41] Similarly, the overexpression of these miRNAs in BF could reflect their involvement in regulating embryonic stemness.

This hypothesis is supported by the dynamic expression profiles of miR-302/367 and miR-371/373 clusters, which increase during the critical window of blastocyst formation and lineage differentiation [19]. Despite the statistically significant up-regulation of eleven BF miRNAs and their potential relevance in stemness, the most remarkable data was that six of them (miR-106a, miR136, miR-203, miR-367, miR-373, and miR-520d-5p) exhibited co-expression, with an *r*-value ≥ 0.7 (Figs. 3A and 4A). Their consistent expression pattern across individual samples makes them particularly intriguing. Therefore, we focused our subsequent analysis on these six miRNAs (Figs. 3B and 4B). While numerous studies have explored the correlation between miRNAs, mRNAs, or protein targets, only a limited number have reported miRNA-miRNA co-expression [42, 43]. Clustered miRNAs are often regulated by a common promoter and can be co-expressed as polycistronic primary transcripts (pri-miRNAs). Alternatively, miRNA-miRNA co-expression could also depend on their involvement in similar biological functions.

Genomic analysis revealed no clustering among the co-expressed miRNAs. Only miR-520b, miR-520d-5p, and miR-525-3p have been found to belong to the primate-specific chromosome 19 miRNA cluster (Table 2). While miR-520b showed a significant correlation and an *r* ≥ 0.7 with miR-520d only in implanted embryos, miR-525 did not show any significant correlation with miR-520b and miR-520d (Fig. 3A). Gene Ontology (GO) analyses, focusing on biological processes (BPs), revealed that miR-106a, miR136, miR-367, miR-373, and miR-520d are significantly associated with 69 common processes, and in Fig. 4B, we show the most interesting ones. In addition, signaling pathway analysis revealed that our miRNAs play a role in regulating cell cycle, cell proliferation, and pluripotency (Fig. 5A). Previous studies have reported altered expression of genes that are involved in cell cycle, cell signaling, and energy metabolic pathways in implantation-incompetent dormant mice and human blastocysts [44, 45].

Furthermore, the participation of the six BF miRNAs in similar biological functions is supported by the fact that some of them share several mRNA targets and that their synthesis is regulated by common transcription factors (TFs). MiR-106, miR-203, and miR-373 are central nodes in the miRNA-mRNA network interaction, and they regulate common mRNAs (Fig. 6). Mir-106 and miR-203 regulate E2F transcription factor 1 (E2F1) and ATM serine/threonine kinase (ATM), controlling, respectively, G1/S and G2/M cell cycle checkpoints [46–48]**.** miR-203 and miR-373 targeting phosphatidylinositol-4,5-bisphosphate 3-kinase catalytic subunit alpha (PIK3CA) can regulate cell proliferation by the AKT/PI3K signaling pathway [48]**.** MYC proto-oncogene, bHLH transcription factor (MYC), NANOG, SOX2, and POU5F1 are TFs regulating the pluripotency by different mechanisms ranging from positive control of cell cycle and metabolism, repression of cell differentiation, and interaction with chromatin modifiers as shown in several studies in humans [49–51] and mice [52–57]**.** Moreover, they extend their regulatory action by integrating complex networks of non-coding RNAs (ncRNAs) [58, 59]. MYC regulates the transcription of miR-106a-5p, miR-203a-3p, and miR-373-3p, and SOX2 regulates the transcription of miR-106a-5p, miR-367-3p, and miR-373-3p. Both NANOG and POU5F1 regulate miR-367-3p and 373-3p (Fig. 7A). The network showing the TF-miRNA-mRNA targets interactions revealed some important regulatory loops demonstrating the common biological function of the six miRNAs (Fig. 7B). Interestingly, MYC regulates the transcription of the miRNAs that share common targets (Fig. 7B). These results strongly suggest that the identified miRNAs, acting in concert, perform an essential role during preimplantation development.

Finally, ROC curve analysis confirmed the predictive value of miR-106a, miR-136, miR-367, miR-373, and miR-520d as potential classifiers of implantation, with statistically significant AUC values ranging between 0.6 and 0.7 (Fig. 8). The predictive ability of miR-203 is not significant, although the AUC, sensitivity, and specificity values of the biomarker remain comparable to those of other miRNAs. Youden’s index calculation provided the optimal threshold value, offering a simple tool for clinical translation [30]. Logistic regression of ROC curves demonstrated improved diagnostic accuracy when considering all six correlated miRNAs together (Fig. 8B). The AUC values did not reach 0.8, likely due to moderate individual variability in expression levels observed in the analyzed samples (Figs. 1 and 3B).

## Conclusions

In summary, the results obtained in this paper identified miR-106a, miR-136, miR-203, miR-367, miR-373, and miR-520d as the epigenetic signature present in BF able to predict embryo implantation potential. A recent study by Chousal et al. identified a genome-wide list of factors and pathways in human blastocysts with good and poor morphology that influence implantation success. Development and integration of transcriptome analysis methods in human blastocysts are critical for improving embryo selection and increasing implantation rates [45]**.** Here, we demonstrated that these miRNAs are highly expressed in implanted embryos compared to non-implanted ones and verified their involvement in stemness and pluripotency pathways within embryo cells by computational analysis. We propose that integrating this miRNA signature with the analysis of transcriptome and embryo ploidy or specific genetic defects could better detect embryos with higher implantation potential. In fact, the genetic background may not necessarily correlate with miRNA profiles and successful implantation, as evidenced by the failure of euploid and high-quality embryos to implant [60].

Many researchers focused their attention on non-invasive potential biomarkers in SCM [61–63]. We believe that the miRNAs present in SCM and BF may serve different functions; the first in mediating the dialog between TE and maternal tissues, while the second, as we demonstrated, plays a role in stemness and pluripotency within embryo cells. Even if blastocentesis is more invasive and requires a higher level of skill and time from the embryologist, BF could better represent the embryo’s secretome being less influenced by external factors [33].

Beyond the potential implications for clinical practice in the field of assisted reproduction, particularly in identifying embryos with the highest implantation potential, our data highlight the fundamental role of microRNAs in regulating stemness and differentiation processes, as well as their critical involvement in mediating cellular communication during embryonic development [64]. Utilizing advanced technologies capable of directly detecting and quantifying miRNAs from various body fluids can facilitate data transfer and streamline analysis processes [65]. This approach holds promise for improving the accuracy and efficiency of embryo selection in assisted reproductive technologies.

## Supplementary Information

Below is the link to the electronic supplementary material.

Supplementary Material 1 (DOCX 17.5 KB)

Supplementary Material 2 (XLSX 68.7 KB)

Supplementary Material 3 (XLSX 11.5 KB)

Supplementary Material 4 (DOCX 16.6 KB)

Supplementary Material 5 (DOCX 13.8 KB)

## Data Availability

The datasets used and/or analyzed during the current study are available from the corresponding author upon reasonable request.
