# Supplementary material for: Up-regulated microRNAs in blastocoel fluid of human implanted embryos could control circuits of pluripotency and be related to embryo competence
Source: J Assist Reprod Genet. 2025 Mar 26;42(5):1635–49. doi: 10.1007/s10815-025-03457-x (PMC12167203; doi:10.1007/s10815-025-03457-x)
Supplement: Supplementary file 1 — Supplementary Material 1 (DOCX 17.5 KB) [file 10815_2025_3457_MOESM1_ESM.docx]

**Table S1.** Custom Card configuration

| Detector | Assay | ID | Type |
| --- | --- | --- | --- |
| A1 | ath-miR159a-000338 | 338 | Negative Control |
| A2 | hsa-miR-106a-002169 | 2169 | Endogenous |
| A3 | hsa-miR-106b-000442 | 442 | Endogenous |
| A4 | hsa-miR-125a-5p-002198 | 2198 | Endogenous |
| A5 | hsa-miR-126-002228 | 2228 | Endogenous |
| A6 | hsa-miR-130a-000454 | 454 | Endogenous |
| A7 | hsa-miR-132-000457 | 457 | Endogenous |
| A8 | hsa-miR-136-000592 | 592 | Endogenous |
| A9 | hsa-miR-138-002284 | 2284 | Endogenous |
| A10 | hsa-miR-141-000463 | 463 | Endogenous |
| A11 | U6 snRNA-001973 | 1973 | Housekeeping |
| A12 | U6 snRNA-001973 | 1973 | Housekeeping |
| A13 | hsa-miR-142-3p-000464 | 464 | Endogenous |
| A14 | hsa-miR-146a-000468 | 468 | Endogenous |
| A15 | hsa-miR-146b-001097 | 1097 | Endogenous |
| A16 | hsa-miR-150-000473 | 473 | Endogenous |
| A17 | hsa-miR-155-002623 | 2623 | Endogenous |
| A18 | hsa-miR-15b-000390 | 390 | Endogenous |
| A19 | hsa-miR-16-000391 | 391 | Endogenous |
| A20 | hsa-miR-17-002308 | 2308 | Endogenous |
| A21 | hsa-miR-184-000485 | 485 | Endogenous |
| A22 | hsa-miR-191-002299 | 2299 | Endogenous |
| A23 | hsa-miR-192-000491 | 491 | Endogenous |
| A24 | hsa-miR-193b-002367 | 2367 | Endogenous |
| B1 | hsa-miR-194-000493 | 493 | Endogenous |
| B2 | hsa-miR-195-000494 | 494 | Endogenous |
| B3 | hsa-miR-197-000497 | 497 | Endogenous |
| B4 | hsa-miR-19a-000395 | 395 | Endogenous |
| B5 | hsa-miR-19b-000396 | 396 | Endogenous |
| B6 | hsa-miR-200c-002300 | 2300 | Endogenous |
| B7 | hsa-miR-202-002363 | 2363 | Endogenous |
| B8 | hsa-miR-203-000507 | 507 | Endogenous |
| B9 | hsa-miR-204-000508 | 508 | Endogenous |
| B10 | hsa-miR-205-000509 | 509 | Endogenous |
| B11 | hsa-miR-20a-000580 | 580 | Endogenous |
| B12 | hsa-miR-20b-001014 | 1014 | Endogenous |
| B13 | hsa-miR-212-000515 | 515 | Endogenous |
| B14 | hsa-miR-222-002276 | 2276 | Endogenous |
| B15 | hsa-miR-223-002295 | 2295 | Endogenous |
| B16 | hsa-miR-24-000402 | 402 | Endogenous |
| B17 | hsa-miR-26a-000405 | 405 | Endogenous |
| B18 | hsa-miR-28-3p-002446 | 2446 | Endogenous |
| B19 | hsa-miR-29a-002112 | 2112 | Endogenous |
| B21 | hsa-miR-301-000528 | 528 | Endogenous |
| B20 | hsa-miR-29c-000587 | 587 | Endogenous |
| B22 | hsa-miR-302a-000529 | 529 | Endogenous |
| B23 | hsa-miR-302b-000531 | 531 | Endogenous |
| B24 | hsa-miR-302c-000533 | 533 | Endogenous |
| C1 | hsa-miR-30b-000602 | 602 | Endogenous |
| C2 | hsa-miR-30c-000419 | 419 | Endogenous |
| C3 | hsa-miR-31-002279 | 2279 | Endogenous |
| C4 | hsa-miR-320-002277 | 2277 | Endogenous |
| C5 | hsa-miR-342-3p-002260 | 2260 | Endogenous |
| C6 | hsa-miR-345-002186 | 2186 | Endogenous |
| C7 | hsa-miR-34a-000426 | 426 | Endogenous |
| C8 | hsa-miR-362-001273 | 1273 | Endogenous |
| C9 | hsa-miR-367-000555 | 555 | Endogenous |
| C10 | hsa-miR-371-3p-002124 | 2124 | Endogenous |
| C11 | hsa-miR-372-000560 | 560 | Endogenous |
| C12 | hsa-miR-373-000561 | 561 | Endogenous |
| C13 | hsa-miR-374-000563 | 563 | Endogenous |
| C14 | hsa-miR-381-000571 | 571 | Endogenous |
| C15 | hsa-miR-410-001274 | 1274 | Endogenous |
| C16 | hsa-miR-454-002323 | 2323 | Endogenous |
| C17 | hsa-miR-484-001821 | 1821 | Endogenous |
| C18 | hsa-miR-512-3p-001823 | 1823 | Endogenous |
| C19 | hsa-miR-515-3p-002369 | 2369 | Endogenous |
| C20 | hsa-miR-515-5p-001112 | 1112 | Endogenous |
| C21 | hsa-miR-517a-002402 | 2402 | Endogenous |
| C22 | hsa-miR-517b-001152 | 1152 | Endogenous |
| C23 | hsa-miR-517c-001153 | 1153 | Endogenous |
| C24 | hsa-miR-518a-3p-002397 | 2397 | Endogenous |
| D1 | hsa-miR-518b-001156 | 1156 | Endogenous |
| D2 | hsa-miR-518d-001159 | 1159 | Endogenous |
| D3 | hsa-miR-518e-002395 | 2395 | Endogenous |
| D4 | hsa-miR-518f-002388 | 2388 | Endogenous |
| D5 | hsa-miR-519a-002415 | 2415 | Endogenous |
| D6 | hsa-miR-519d-002403 | 2403 | Endogenous |
| D7 | hsa-miR-520a#-001168 | 1168 | Endogenous |
| D8 | hsa-miR-520b-001116 | 1116 | Endogenous |
| D9 | hsa-miR-520d-5p-002393 | 2393 | Endogenous |
| D10 | hsa-miR-520g-001121 | 1121 | Endogenous |
| D11 | hsa-miR-522-002413 | 2413 | Endogenous |
| D12 | hsa-miR-525-3p-002385 | 2385 | Endogenous |
| D13 | hsa-miR-526b-002382 | 2382 | Endogenous |
| D14 | hsa-miR-532-001518 | 1518 | Endogenous |
| D15 | hsa-miR-548a-001538 | 1538 | Endogenous |
| D16 | hsa-miR-548c-001590 | 1590 | Endogenous |
| D17 | hsa-miR-590-5p-001984 | 1984 | Endogenous |
| D18 | hsa-miR-597-001551 | 1551 | Endogenous |
| D19 | hsa-miR-886-3p-002194 | 2194 | Endogenous |
| D20 | hsa-miR-886-5p-002193 | 2193 | Endogenous |
| D21 | hsa-miR-9-000583 | 583 | Endogenous |
| D22 | hsa-miR-92a-000431 | 431 | Endogenous |
| D23 | RNU44-001094 | 1094 | Positive Control |
| D24 | RNU48-001006 | 1006 | Positive Control |
