# Supplementary material for: Up-regulated microRNAs in blastocoel fluid of human implanted embryos could control circuits of pluripotency and be related to embryo competence
Source: J Assist Reprod Genet. 2025 Mar 26;42(5):1635–49. doi: 10.1007/s10815-025-03457-x (PMC12167203; doi:10.1007/s10815-025-03457-x)
Supplement: Supplementary file 4 — Supplementary Material 4 (DOCX 16.6 KB) [file 10815_2025_3457_MOESM4_ESM.docx]

**Table S4.** Transcription factors interacting with at least two out of six DE miRNAs.

| **All TF** | **miR-106a-5p** | **miR-136-5p** | **miR-203a-3p** | **miR-367-3p** | **miR-373-3p** | **miR-520d** |
| --- | --- | --- | --- | --- | --- | --- |
| AR |  |  |  |  |  |  |
| BRD3 |  |  |  |  |  |  |
| E2F1 |  |  |  |  |  |  |
| E2F6 |  |  |  |  |  |  |
| EGR1 |  |  |  |  |  |  |
| EP300 |  |  |  |  |  |  |
| ERG |  |  |  |  |  |  |
| ESR1 |  |  |  |  |  |  |
| FOXA1 |  |  |  |  |  |  |
| GABPA |  |  |  |  |  |  |
| GTF2I |  |  |  |  |  |  |
| MAX |  |  |  |  |  |  |
| MYC |  |  |  |  |  |  |
| NRF1 |  |  |  |  |  |  |
| OTX2 |  |  |  |  |  |  |
| SOX2 |  |  |  |  |  |  |
| SP1 |  |  |  |  |  |  |
| SUMO2 |  |  |  |  |  |  |
| ZNF143 |  |  |  |  |  |  |
| CEBPB |  |  |  |  |  |  |
| CREB1 |  |  |  |  |  |  |
| HIF1A |  |  |  |  |  |  |
| STAT3 |  |  |  |  |  |  |
| TEAD4 |  |  |  |  |  |  |
| CTNNB1 |  |  |  |  |  |  |
| EOMES |  |  |  |  |  |  |
| LEF1 |  |  |  |  |  |  |
| NANOG |  |  |  |  |  |  |
| POU5F1 |  |  |  |  |  |  |
