# Supplementary material for: Up-regulated microRNAs in blastocoel fluid of human implanted embryos could control circuits of pluripotency and be related to embryo competence
Source: J Assist Reprod Genet. 2025 Mar 26;42(5):1635–49. doi: 10.1007/s10815-025-03457-x (PMC12167203; doi:10.1007/s10815-025-03457-x)
Supplement: Supplementary file 5 — Supplementary Material 5 (DOCX 13.8 KB) [file 10815_2025_3457_MOESM5_ESM.docx]

**Table S5.** Transcription factors interacting with at least two out of six DE miRNAs that share common mRNA target genes.

| **Transcription Factors** | **miRNA** | **Target Gene** |
| --- | --- | --- |
| AR, E2F1, EGR1, EP300, ERG, ESR1, MAX, MYC, NRF1, SP1, SUMO2, ZNF143 | miR-106a-5p | ATM, E2F1 |
|  | miR-203a-3p |  |
| EP300, MYC, SP1, HIF1A | miR-203a-3p | PIK3CA |
|  | miR-373-3p |  |
| EP300, GABPA, MYC, OTX2, SOX2, SP1 | miR-106a-5p | TGFBR2 |
|  | miR-373-3p |  |
| EP300, MYC, SP1 | miR-106a-5p | VEGFA |
|  | miR-203a-3p |  |
|  | miR-373-3p |  |
